# Supplementary material for: Asparagine Metabolism in Tumors Is Linked to Poor Survival in Females with Colorectal Cancer: A Cohort Study
Source: Metabolites. 2022 Feb 9;12(2):164. doi: 10.3390/metabo12020164 (PMC8875032; doi:10.3390/metabo12020164)
Supplement: Supplementary file 1 [file metabolites-12-00164-s001.zip › metabolites-1566669-supplementary.pdf]

Supplementary materials

Table S1. Demographic characteristics and clinical factors for each subgroup.

Subgroup		Stage I (n=47)		Stage II (n=86)		Stage III (n=64)	
		RCC (n=22)	LCC (n=25)	RCC (n=44)	LCC (n=42)	RCC (n=32)	LCC (n=32)
**Sex, n**	Males	10	15	23	25	15	14
	Females	12	10	21	17	17	18
**Age, y, mean (SD)**	Males	73.9 (6.5)	69.3 (5.8)	72.9 (7.8)	72.2 (8.5)	73.5 (7.8)	63.7 (5.8)
	Females	72.1 (6.2)	69.6 (7.6)	73.5 (9.8)	69.1 (7.8)	72.2 (6.6)	71.1 (6.0)
**Race/Ethnicity, n**	NHWs	20	22	40	37	26	25
	Hispanic	2	3	2	3	4	2
	AA	0	0	1	0	1	4
	API	0	0	1	2	1	1
***KRAS* mutation, n**	Wild type	7	14	24	21	16	19
	Mutant	8	8	12	13	10	9
	Unknown	7	3	8	8	6	4
***BRAF* mutation, n**	Wild type	13	19	20	30	18	27
	Mutant	0	0	9	1	8	0
	Unknown	9	6	15	11	6	5
**Microsatellite instability status, n**	MSS	10	16	17	27	15	17
	MSI	5	4	17	4	10	8
	Unknown	7	5	10	11	7	7
**5-year Overall survival rate, %[a]**	Males	87.5	85.1	76.5	74.1	47.1	78.6
	Females	100	100	82.9	100	65.2	61.8
**5-year Recurrence-free survival rate, %[a]**	Males	90.0	78.3	86.8	67.6	82.1	70.1
	Females	90.9	100.0	87.5	87.4	76.0	68.8

RCC: right-sided colon cancer, LCC: left-sided colon cancer, NHWs: non-Hispanic whites, AA: African-Americans, API: Asian-Pacific Islander, MSI; microsatellite instable, MSS; microsatellite stable. [a] The survival rates were calculated using the Kaplan-Meier estimation method.

Table S2. Prognosis of patients in early stage (Stage I and II) vs late stage (Stage III).

5-year Overall Survival (OS)								
**Subgroup**	**RCC males**		**RCC females**		**LCC males**		**LCC females**	
**Event[a]**	**0**	**1**	0	1	0	1	0	1
**Early stage[b]**	28	5	30	3	32	8	27	0
**Late stage[c]**	8	7	12	5	11	3	12	6
**Total**	36	12	42	8	43	11	39	6
**5-year Recurrence-free Survival (RFS)**								
**Subgroup**	**RCC males**		**RCC females**		**LCC males**		**LCC females**	
**Event[d]**	0	1	0	1	0	1	0	1
**Early stage[b]**	30	3	30	3	32	8	25	2
**Late stage[c]**	13	2	14	3	10	4	13	5
**Total**	43	5	44	6	42	12	38	7

[a] Event = death. Event =1 represents that death occurred within 5 years of follow-up, and event = 0 indicates the event did not occur within 5 years of follow-up (survived or scored as censored). [b] Early stage combines stage I and II together. [c] Late stage refers to stage III. [d] Event = recurrence. Event = 1 indicates that recurrence occurred within 5 years of follow-up, event = 0 indicates that recurrence did not occur within 5 years of follow-up. If a patient died before CRC recurrence occurred, it is counted towards a death event.

Notes:

If a patient died for any reason without recurrence within 5 years of follow-up, it is only counted toward a death event in OS.

If a patient experienced recurrence but did not die within 5 years of follow-up, it is only counted toward a recurrence event in RFS.

If a patient experienced recurrence and then died within 5 years of follow-up, it is counted toward a recurrence event in RFS and a death event in OS.

If a patient experienced neither recurrence nor death within 5 years of follow-up, the event is 0 for both OS and RFS.

Due to the absence of death events in females of clinical stage I, we regarded stage I and II as early stage, and stage III as late stage.

Table S3. Multivariate analyses of the associations between individual metabolites and 5-year OS by sex, adjusted for anatomic location, chemotherapy history, clinical stages, and age.

Metabolite name	Females			Males			Int. Sex *P* value[b]
	HR	95% CI	*P* value[a]	HR	95% CI	*P* value[a]	
**Acetyl-lysine**	0.97	0.75–1.25	0.809	0.82	0.71–0.96	0.012	0.298
Adenosine	0.90	0.69–1.17	0.421	1.29	1.03–1.62	0.026	0.038
Alanine	1.06	0.78–1.44	0.705	0.77	0.61–0.98	0.034	0.092
Argininosuccinic acid	0.93	0.71–1.23	0.629	0.74	0.58–0.93	0.010	0.151
Asparagine	1.51	0.89–2.57	0.127	0.72	0.54–0.96	0.025	0.026
Carnitine	0.61	0.04–9.10	0.717	0.53	0.30–0.94	0.031	0.897
Citrulline	1.66	0.97–2.83	0.065	0.65	0.46–0.92	0.014	0.002
Glycerol 3-phosphate	3.64	1.26–10.49	0.017	0.91	0.47–1.77	0.777	0.018
Hypoxanthine	1.05	0.35–3.14	0.929	0.65	0.44–0.95	0.027	0.441
LysoPC(16:0)	1.52	1.03–2.25	0.035	0.85	0.65–1.11	0.242	0.009
Ornithine	0.97	0.56–1.66	0.904	0.68	0.47–0.97	0.035	0.310
Serine	1.25	0.65–2.41	0.497	0.55	0.37–0.81	0.002	0.035
Spermine	1.49	1.04–2.13	0.031	1.03	0.83–1.27	0.811	0.073
Succinate	0.35	0.12–0.99	0.047	1.91	1.23–2.96	0.004	0.004
Threonine	1.12	0.67–1.87	0.670	0.61	0.44–0.85	0.004	0.035
UDP-D-Glucose	0.81	0.67–0.97	0.025	1.16	0.95–1.41	0.152	0.010
Uracil	1.24	0.56–2.76	0.599	0.42	0.26–0.69	0.001	0.024
Xanthosine	1.22	0.86–1.73	0.266	0.71	0.54–0.94	0.016	0.028

[a] *P* values for the association between individual metabolites and 5-year OS calculated by multivariate Cox PH regression models, adjusted for anatomic location, clinical stages, chemotherapy history and age (before FDR adjustment). The abundance of each metabolite was treated as a continuous variable and was $\log_2$ transformed. A metabolite with HR < 1 was associated with better OS; a metabolite with HR > 1 was associated with worse OS. [b] *P* value of the interaction between the metabolite and sex. Int. Sex *P* value < .05 indicate a significant sex interaction.

Table S4. Multivariate analyses of the associations between individual metabolites and 5-year RFS by sex, adjusted for anatomic location, chemotherapy history, clinical stages, and age.

Metabolite name	Females			Males			Int. Sex *P* value[b]
	HR	95% CI	*P* value[a]	HR	95% CI	*P* value[a]	
**Acetyl-lysine**	0.98	0.76–1.26	0.855	0.78	0.67–0.92	0.002	0.076
Alanine	1.12	0.81–1.55	0.495	0.74	0.58–0.94	0.013	0.053
AMP	1.10	0.69–1.77	0.689	0.69	0.51–0.95	0.021	0.124
Argininosuccinic acid	1.03	0.79–1.35	0.825	0.70	0.54–0.92	0.011	0.036
Asparagine	1.54	0.93–2.56	0.093	0.74	0.56–0.97	0.031	0.007
Carnitine	3.52	0.25–50.41	0.354	0.37	0.20–0.66	0.001	0.117
CMP	0.97	0.57–1.65	0.906	0.70	0.50–0.96	0.029	0.287
Creatinine	0.75	0.42–1.32	0.317	1.70	1.15–2.51	0.008	0.040
Cytidine	0.46	0.28–0.75	0.002	0.75	0.49–1.16	0.194	0.134
Fructose 6-phosphate	0.84	0.55–1.28	0.414	0.68	0.49–0.95	0.026	0.534
Glutamine	0.77	0.26–2.30	0.642	0.47	0.23–0.97	0.041	0.338
Glutathione	0.73	0.56–0.95	0.019	0.92	0.79–1.06	0.246	0.302
Glutathione disulfide	0.72	0.57–0.91	0.006	0.81	0.67–0.98	0.034	0.092
GMP	1.04	0.69–1.58	0.838	0.74	0.57–0.96	0.024	0.169
Hypoxanthine	1.95	0.59–6.45	0.276	0.32	0.20–0.51	<0.001	0.008
LysoPC(16:1)	1.06	0.76–1.49	0.729	0.77	0.61–0.97	0.025	0.106
LysoPE(18:2)	1.07	0.70–1.65	0.750	0.72	0.55–0.96	0.023	0.127
LysoPE(20:1)	0.98	0.77–1.24	0.856	0.83	0.70–0.98	0.031	0.225
LysoPE(22:5)	1.09	0.70–1.70	0.701	0.70	0.53–0.92	0.010	0.072
Serine	1.45	0.74–2.84	0.282	0.57	0.40–0.83	0.003	0.010
Sphinganine-1-phosphate	0.94	0.60–1.47	0.777	0.66	0.45–0.95	0.026	0.216
Stearamide	0.92	0.63–1.36	0.674	0.70	0.53–0.92	0.012	0.211
Threonine	0.98	0.58–1.64	0.929	0.64	0.46–0.89	0.007	0.074
Xanthine	0.62	0.38–1.01	0.053	0.69	0.48–0.98	0.036	0.869
Xanthosine	1.01	0.72–1.40	0.973	0.71	0.52–0.98	0.040	0.112

[a] *P* values for the association between individual metabolites and 5-year RFS calculated by multivariate Cox PH regression models, adjusted for anatomic location, clinical stages, chemotherapy history and age (before FDR adjustment). The abundance of each metabolite was treated as a continuous variable and was $\log_2$ transformed. A metabolite with HR < 1 was associated with better RFS; a metabolite with HR > 1 was associated with worse RFS. [b] *P* value of the interaction between the metabolite and sex. Int. Sex *P* value < .05 indicate a significant sex interaction.

Table S5. Multivariate analysis of association between metabolites in ASNS-catalyzed asparagine synthesis pathway and OS and RFS for all patients .

Variable[a]	OS				RFS			
	HR	95% CI	*P* value	Int. Sex *P* value[b]	HR	95% CI	*P* value	Int. Sex *P* value[b]
**Asparagine**	**0.89**	**0.61–1.29**	**0.535**	**0.021**	**1.27**	**0.84–1.92**	**0.251**	0.003
Aspartate	1.25	0.85–1.84	0.257	0.639	1.31	0.86–2.00	0.202	0.237
Glutamate	0.93	0.45–1.90	0.837	0.808	0.93	0.44–1.97	0.841	0.064
Glutamine	0.86	0.35–2.08	0.734	0.974	0.37	0.16–0.87	0.023	0.285
AMP	0.83	0.64–1.06	0.135	0.860	0.75	0.57–0.98	0.034	0.295
Sex = Male (ref: Female)	2.01	1.00–4.05	0.051	–	1.12	0.53–2.38	0.761	–
Anatomic location = RCC (ref: LCC)	0.87	0.43–1.77	0.710	–	0.67	0.31–1.44	0.300	–
Clinical stage = late (ref: early)	4.48	2.26–9.24	0.003	–	1.91	0.63–5.79	0.252	–
Chemotherapy = Yes (ref: No)	1.03	0.38–2.76	0.956	–	1.26	0.41–3.86	0.685	–
Age	1.10	1.05–1.15	<0.001	–	0.97	0.92–1.03	0.304	–

[a] All the variables listed were included in one multivariate Cox PH model where the abundance of each metabolite was treated as a continuous variable and was log_2 transformed. [b] *P* value of the interaction between the metabolite and sex.

Table S6. Multivariate analysis of association between metabolites in ASNS-catalyzed asparagine synthesis metabolic pathway and OS and RFS by sex.

Variable[a]	OS						RFS					
	Females			Males			Females			Males		
	HR	95% CI	*P* value	HR	95% CI	*P* value	HR	95% CI	*P* value	HR	95% CI	*P* value
Asparagine	6.39	1.78–22.91	0.004	0.57	0.36–0.91	0.018	4.36	1.39–13.68	0.012	0.96	0.61–1.50	0.856
Aspartate	1.47	0.64–3.40	0.365	1.32	0.75–2.32	0.339	1.60	0.71–4.05	0.239	1.40	0.79–2.50	0.252
Glutamate	0.24	0.06–0.95	0.042	1.28	0.48–3.38	0.618	0.76	0.19–3.11	0.705	0.75	0.28–2.02	0.573
Glutamine	0.16	0.03–1.06	0.057	1.18	0.39–3.54	0.772	0.10	0.02–0.57	0.010	0.52	0.17–1.63	0.266
AMP	0.65	0.31–1.37	0.252	0.89	0.65–1.22	0.476	0.59	0.25–1.36	0.214	0.70	0.49–1.00	0.049
Anatomic location = RCC (ref: LCC)	1.63	0.47–5.65	0.438	0.80	0.31–2.10	0.653	0.78	0.23–2.65	0.692	0.61	0.20–1.84	0.380
Clinical stage = late (ref: early)	14.90	2.19–101.43	0.006	6.02	1.39–26.06	0.016	1.92	0.34–10.99	0.462	1.29	0.23–7.09	0.771
Chemotherapy = Yes (ref: No)	1.78	0.39–8.23	0.458	0.74	0.18–2.99	0.676	2.34	0.38–14.29	0.358	1.01	0.19–5.24	0.991
Age	1.09	1.00–1.19	0.037	1.15	1.07–1.23	<0.001	1.01	0.91–1.11	0.905	0.94	0.87–1.01	0.112

[a] All the variables listed were included in one multivariate Cox PH model. The abundance of each metabolite was treated as a continuous variable and was $\log_2$ transformed.

Table S7. Multivariate analysis of association between metabolites in PPP and glycolysis metabolic pathway and OS and RFS for all patients .

Variable[a]	OS				RFS			
	HR	95% CI	*P* value[a]	Int. Sex *P* value[b]	HR	95% CI	*P* value[a]	Int. Sex *P* value[b]
**Ribulose 5-phosphate**	**1.18**	**0.84–1.65**	**0.354**	**0.934**	**1.33**	**0.89–1.99**	**0.164**	0.263
Lactate	1.49	0.81–2.75	0.203	0.601	1.21	0.51–2.85	0.660	0.472
DHAPorG3P	1.21	0.72–2.03	0.479	0.214	0.99	0.57–1.71	0.975	0.137
Glucose 6-phosphate	0.95	0.67–1.35	0.775	0.817	1.30	0.90–1.87	0.156	0.110
Fructose 6-phosphate	0.68	0.42–1.10	0.114	0.899	0.46	0.27–0.78	0.004	0.615
Glycerol 3-phosphate	1.44	0.80–2.59	0.222	0.046	0.53	0.25–1.12	0.097	0.253
Phosphoenolpyruvate	1.05	0.83–1.35	0.670	0.241	1.17	0.84–1.61	0.358	0.418
Sex = Male (ref: Female)	1.95	0.97–3.90	0.060	–	1.33	0.64–2.78	0.442	–
Anatomic location = RCC (ref: LCC)	0.65	0.29–1.42	0.279	–	0.83	0.36–1.93	0.665	–
Clinical stage = late (ref: early)	4.24	1.58–11.42	0.004	–	1.53	0.46–5.14	0.491	–
Chemotherapy = Yes (ref: No)	1.01	0.36–2.85	0.980	–	1.63	0.47–5.72	0.442	–
Age	1.11	1.06–1.16	<0.001	–	0.99	0.93–1.04	0.596	–

[a] All the variables listed were included in one multivariate Cox PH model where the abundance of each metabolite was treated as a continuous variable and was $\log_2$ transformed. [b] *P* value of the interaction between the metabolite and sex.

Table S8. Multivariate analysis of association between metabolites in PPP and glycolysis metabolic pathway and OS and RFS by sex.

Variable[a]	OS				RFS			
	Females		Males		Females		Males	
	HR (95% CI)	*P* value	HR (95% CI)	*P* value	HR (95% CI)	*P* value	HR (95% CI)	*P* value
Ribulose 5-phosphate	1.30 (0.72–2.35)	0.388	1.09 (0.70–1.69)	0.700	1.87 (0.96–3.65)	0.068	1.13 (0.63–2.03)	0.673
Lactate	1.63 (0.45–5.98)	0.460	1.02 (0.45–2.34)	0.960	1.17 (0.26–5.18)	0.839	0.92 (0.28–3.00)	0.885
DHAPorG3P	2.20 (0.75–6.48)	0.153	0.91 (0.48–1.75)	0.785	1.35 (0.51–3.56)	0.550	0.94 (0.45–1.98)	0.879
Glucose 6-phosphate	0.93 (0.48–1.81)	0.842	0.86 (0.51–1.43)	0.553	1.90 (1.00–3.58)	0.048	0.74 (0.44–1.24)	0.254
Fructose 6-phosphate	0.77 (0.32–1.87)	0.570	0.96 (0.50–1.82)	0.890	0.26 (0.11–0.64)	0.003	0.72 (0.37–1.42)	0.344
Glycerol 3-phosphate	2.94 (0.86–9.99)	0.084	0.89 (0.42–1.88)	0.760	0.79 (0.27–2.28)	0.664	0.27 (0.08–0.88)	0.029
Phosphoenolpyruvate	1.16 (0.69–1.93)	0.572	0.96 (0.70–1.33)	0.817	1.54 (0.85–2.77)	0.154	0.98 (0.63–1.53)	0.940
Anatomic location = RCC (ref: LCC)	1.01 (0.27–3.80)	0.993	0.58 (0.17–1.91)	0.367	1.03 (0.27–3.94)	0.968	0.80 (0.23–2.78)	0.728
Clinical stage = late (ref: early)	13.00 (1.96–86.35)	0.008	3.36 (0.87–13.02)	0.080	2.00 (0.20–19.67)	0.551	0.99 (0.18–5.34)	0.994
Chemotherapy = Yes (ref: No)	0.75 (0.13–4.43)	0.753	0.99 (0.23–4.20)	0.987	1.65 (0.16–16.89)	0.675	1.21 (0.24–6.12)	0.816
Age	1.08 (0.98–1.20)	0.132	1.12 (1.05–1.19)	0.001	1.01 (0.92–1.10)	0.832	0.92 (0.84–1.00)	0.064

[a] All the variables listed were included in one multivariate Cox PH model. The abundance of each metabolite was treated as a continuous variable and was $\log_2$ transformed.

Table S9. Multivariate analysis of association between metabolites in lysophospholipids synthesis and OS and RFS for all patients .

**Variable**[a]	**OS**			**RFS**		
	**HR (95% CI)**	***P* value**[a]	**Int. Sex *P* value**[b]	**HR (95% CI)**	***P* value**[a]	**Int. Sex *P* value**[b]
**Oleic acid**	**1.13 (0.82–1.56)**	**0.457**	**0.271**	**1.33 (0.94–1.89)**	**0.107**	0.472
L-Palmitoylcarnitine	1.25 (0.76–2.06)	0.378	0.784	2.64 (1.43–4.86)	0.002	0.481
Stearoylcarnitine	0.84 (0.64–1.09)	0.190	0.900	0.76 (0.56–1.04)	0.089	0.595
Carnitine	0.52 (0.22–1.23)	0.137	0.681	0.29 (0.11–0.78)	0.014	0.098
LysoPC(16:0)	1.22 (0.83–1.80)	0.306	0.002	0.87 (0.44–1.72)	0.691	0.233
LysoPC(16:1)	0.68 (0.39–1.20)	0.183	0.153	1.18 (0.61–2.28)	0.626	0.376
LysoPC(18:1)	0.75 (0.40–1.41)	0.364	0.018	1.07 (0.53–2.14)	0.854	0.447
LysoPE(16:0)	1.66 (0.69–3.99)	0.253	0.088	4.38 (1.75–10.97)	0.002	0.443
LysoPE(16:1)	0.98 (0.56–1.72)	0.940	0.675	0.70 (0.33–1.49)	0.358	0.720
LysoPE(18:0)	0.84 (0.37–1.90)	0.674	0.027	1.04 (0.47–2.29)	0.924	0.283
LysoPE(18:1)	0.88 (0.31–2.50)	0.817	0.275	0.36 (0.13–1.00)	0.051	0.595
LysoPE(20:1)	1.08 (0.70–1.65)	0.740	0.205	1.04 (0.64–1.69)	0.875	0.334
LysoPE(22:5)	0.88 (0.41–1.93)	0.758	0.042	0.50 (0.24–1.07)	0.073	0.052
LysoPE(18:2)	1.52 (0.84–2.77)	0.167	0.467	0.99 (0.49–1.98)	0.970	0.395
Sex = Male (ref: Female)	2.30 (1.07–4.92)	0.032	–	1.58 (0.73–3.43)	0.250	–
Anatomic location = RCC (ref: LCC)	0.59 (0.26–1.35)	0.213	–	0.74 (0.28–1.92)	0.532	–
Clinical stage = late (ref: early)	4.02 (1.42–11.38)	0.009	–	3.70 (1.26–10.84)	0.017	–
Chemotherapy = Yes (ref: No)	1.42 (0.48–4.16)	0.523	–	0.91 (0.30–2.72)	0.862	–
Age	1.11 (1.06–1.16)	<0.001	–	0.96 (0.90–1.02)	0.174	–

Abbreviation: LysoPC, lysophosphatidylcholine; LysoPE, lysophosphatidylethanolamine. [a] All the variables listed were included in one multivariate Cox PH model where the abundance of each metabolite was treated as a continuous variable and was $\log_2$ transformed. [b] *P* value of the interaction between the metabolite and sex.

Table S10. Multivariate analysis of association between metabolites in lysophospholipids synthesis and OS and RFS by sex.

Variable[a]	OS				RFS			
	Females		Males		Females		Males	
	HR (95% CI)	*P* value	HR (95% CI)	*P* value	HR (95% CI)	*P* value	HR (95% CI)	*P* value
Oleic acid	1.05 (0.50–2.20)	0.905	1.66 (1.02–2.69)	0.041	2.27 (0.95–5.44)	0.065	0.91 (0.53–1.57)	0.728
L-Palmitoylcarnitine	1.55 (0.61–3.89)	0.355	2.99 (1.03–8.69)	0.044	3.93 (1.29–12.04)	0.016	1.79 (0.57–5.64)	0.323
Stearoylcarnitine	0.65 (0.41–1.05)	0.076	0.60 (0.34–1.05)	0.074	0.60 (0.37–0.99)	0.047	0.75 (0.42–1.34)	0.329
Carnitine	6.83 (0.11–431.17)	0.363	0.19 (0.06–0.67)	0.009	5.84 (0.08–435.95)	0.422	0.19 (0.03–1.03)	0.054
LysoPC(16:0)	2.07 (1.06–4.07)	0.034	0.64 (0.27–1.54)	0.319	1.06 (0.37–3.04)	0.920	0.43 (0.08–2.21)	0.313
LysoPC(16:1)	0.39 (0.11–1.37)	0.144	0.52 (0.22–1.27)	0.151	2.71 (0.66–11.17)	0.169	0.86 (0.27–2.70)	0.798
LysoPC(18:1)	1.56 (0.43–5.75)	0.501	0.29 (0.08–1.00)	0.050	0.41 (0.10–1.65)	0.210	7.82 (1.30–47.16)	0.025
LysoPE(16:0)	1.66 (0.35–7.95)	0.524	12.72 (2.20–73.55)	0.005	1.52 (0.34–6.83)	0.588	6.58 (0.99–43.95)	0.052
LysoPE(16:1)	1.27 (0.38–4.30)	0.695	1.16 (0.50–2.73)	0.726	1.07 (0.26–4.45)	0.927	0.70 (0.18–2.73)	0.604
LysoPE(18:0)	2.56 (0.65–10.11)	0.179	0.10 (0.02–0.44)	0.002	4.21 (0.79–22.39)	0.092	2.64 (0.36–19.31)	0.338
LysoPE(18:1)	0.57 (0.06–5.17)	0.618	0.80 (0.14–4.63)	0.806	0.07 (0.01–0.63)	0.017	0.30 (0.04–2.19)	0.233
LysoPE(20:1)	0.71 (0.25–2.04)	0.521	1.14 (0.62–2.11)	0.679	0.33 (0.07–1.47)	0.147	1.37 (0.61–3.06)	0.444
LysoPE(22:5)	0.78 (0.21–2.93)	0.714	2.47 (0.66–9.16)	0.179	5.38 (1.02–28.42)	0.048	0.10 (0.02–0.62)	0.013
LysoPE(18:2)	1.06 (0.20–5.45)	0.948	2.41 (1.12–5.20)	0.024	1.59 (0.31–8.26)	0.580	0.50 (0.14–1.77)	0.286

Anatomic location = RCC (ref: LCC)	1.05 (0.29–3.79)	0.941	0.09 (0.02–0.43)	0.002	1.11 (0.19–6.58)	0.909	1.00 (0.14–6.97)	1.000
Clinical stage = late (ref: early)	18.99 (2.31–155.90)	0.006	15.45 (2.48–96.39)	0.003	4.40 (0.24–79.55)	0.316	2.49 (0.36–17.33)	0.355
Chemotherapy = Yes (ref: No)	0.84 (0.13–5.37)	0.853	0.83 (0.16–4.37)	0.827	2.24 (0.14–36.07)	0.570	0.51 (0.08–3.33)	0.485
Age	1.10 (1.01–1.21)	0.038	1.30 (1.16–1.45)	<0.001	1.05 (0.96–1.16)	0.278	0.86 (0.76–0.97)	0.018

Abbreviation: LysoPC, lysophosphatidylcholine; LysoPE, lysophosphatidylethanolamine. [a] All the variables listed were included in one multivariate Cox PH model. The abundance of each metabolite was treated as a continuous variable and was $\log_2$ transformed.

Table S11. Multivariate analysis of association between metabolites in methionine metabolism and OS and RFS for all patients .

**Variable**[a]	**OS**			**RFS**		
	**HR (95% CI)**	***P* value**[a]	**Int. Sex *P* value**[b]	**HR (95% CI)**	***P* value**[a]	**Int. Sex *P* value**[b]
**Dimethylglycine**	**0.99 (0.64–1.52)**	**0.955**	**0.390**	**0.74 (0.46–1.20)**	**0.228**	0.555
Methionine	1.26 (0.73–2.19)	0.408	0.045	1.90 (1.01–3.57)	0.045	0.022
S-adenosylhomocysteine (SAH)	0.96 (0.75–1.23)	0.751	0.078	0.96 (0.72–1.27)	0.753	0.931
S-adenosylmethionine (SAM)	1.08 (0.88–1.31)	0.467	0.355	1.07 (0.86–1.32)	0.541	0.765
Serine	0.56 (0.34–0.92)	0.023	0.039	0.49 (0.29–0.83)	0.008	0.045
Sex = Male (ref: Female)	2.02 (0.99–4.13)	0.054	–	1.14 (0.53–2.44)	0.740	–
Anatomic location = RCC (ref: LCC)	0.77 (0.38–1.58)	0.483	–	0.62 (0.28–1.37)	0.235	–
Clinical stage = late (ref: early)	5.32 (2.01–14.11)	0.001	–	1.88 (0.60–5.85)	0.277	–
Chemotherapy = Yes (ref: No)	1.02 (0.40–2.61)	0.962	–	1.24 (0.41–3.77)	0.703	–
Age	1.10 (1.05–1.15)	<0.001	–	0.96 (0.91–1.02)	0.189	–

[a] All the variables listed were included in one multivariate Cox PH model where the abundance of each metabolite was treated as a continuous variable and was $\log_2$ transformed. [b] *P* value of the interaction between the metabolite and sex.

Table S12. Multivariate analysis of association between metabolites in methionine metabolism and OS and RFS by sex.

Variable[a]	OS				RFS			
	Females		Males		Females		Males	
	HR (95% CI)	*P* value	HR (95% CI)	*P* value	HR (95% CI)	*P* value	HR (95% CI)	*P* value
Dimethylglycine	0.69 (0.33–1.42)	0.308	1.45 (0.77–2.74)	0.250	0.63 (0.29–1.36)	0.239	0.69 (0.33–1.46)	0.331
Methionine	2.52 (0.27–23.62)	0.419	1.30 (0.64–2.65)	0.465	5.49 (0.49–61.80)	0.168	1.39 (0.62–3.10)	0.423
S-adenosylhomocysteine (SAH)	0.87 (0.56–1.36)	0.545	1.09 (0.76–1.58)	0.631	1.07 (0.67–1.72)	0.776	0.81 (0.54–1.21)	0.304
S-adenosylmethionine (SAM)	1.18 (0.78–1.78)	0.426	1.04 (0.80–1.36)	0.757	1.08 (0.72–1.62)	0.709	1.20 (0.90–1.60)	0.211
Serine	0.48 (0.05–4.59)	0.527	0.37 (0.19–0.72)	0.004	0.31 (0.03–3.13)	0.322	0.49 (0.24–0.98)	0.044
Anatomic location = RCC (ref: LCC)	1.32 (0.42–4.17)	0.636	0.58 (0.22–1.58)	0.289	0.81 (0.26–2.53)	0.714	0.43 (0.12–1.50)	0.184
Clinical stage = late (ref: early)	9.44 (1.44–61.68)	0.019	9.11 (2.22–37.39)	0.002	2.25 (0.42–12.14)	0.345	0.76 (0.13–4.39)	0.764
Chemotherapy = Yes (ref: No)	1.12 (0.24–5.18)	0.886	0.81 (0.23–2.82)	0.742	2.04 (0.39–10.71)	0.398	1.67 (0.34–8.30)	0.529
Age	1.06 (0.98–1.14)	0.134	1.16 (1.08–1.25)	<0.001	0.97 (0.90–1.06)	0.541	0.93 (0.86–1.01)	0.076

[a] All the variables listed were included in one multivariate Cox PH model. The abundance of each metabolite was treated as a continuous variable and was $\log_2$ transformed.

Table S13. Multivariate analysis of association between metabolites in polyamine synthesis and OS and RFS for all patients .

Variable[a]	OS			RFS		
	HR (95% CI)	*P* value[a]	Int. Sex *P* value[b]	HR (95% CI)	*P* value[a]	Int. Sex *P* value[b]
***N*[1], *N*[12]-diacetylspermine (DAS)**	**0.87 (0.62–1.23)**	**0.439**	**0.356**	**1.01 (0.68–1.50)**	**0.954**	0.336
Spermidine	1.30 (0.64–2.63)	0.465	0.594	0.72 (0.34–1.53)	0.396	0.329
Spermine	1.16 (0.83–1.62)	0.386	0.082	1.06 (0.76–1.49)	0.719	0.933
N[1]-acetylspermine	1.07 (0.78–1.48)	0.675	0.887	1.24 (0.89–1.74)	0.203	0.109
Ornithine	0.97 (0.54–1.74)	0.927	0.105	0.84 (0.51–1.39)	0.503	0.206
Arginine	1.38 (0.83–2.27)	0.210	0.070	2.03 (1.14–3.60)	0.016	0.037
Citrulline	0.96 (0.69–1.34)	0.818	0.002	1.05 (0.73–1.51)	0.801	0.023
Argininosuccinic acid	0.69 (0.48–0.98)	0.039	0.123	0.72 (0.49–1.04)	0.081	0.055
Sex = Male (ref: Female)	2.15 (1.08–4.29)	0.030	–	1.17 (0.54–2.56)	0.690	–
Anatomic location = RCC (ref: LCC)	0.81 (0.39–1.71)	0.584	–	0.65 (0.29–1.48)	0.306	–
Clinical stage = late (ref: early)	6.11 (1.91–19.57)	0.002	–	1.93 (0.64–5.81)	0.242	–
Chemotherapy = Yes (ref: No)	0.92 (0.28–2.95)	0.882	–	1.14 (0.36–3.63)	0.824	–
Age	1.10 (1.05–1.15)	<0.001	–	0.96 (0.90–1.01)	0.120	–

[a] All the variables listed were included in one multivariate Cox PH model where the abundance of each metabolite was treated as a continuous variable and was $\log_2$ transformed. [b] *P* value of the interaction between the metabolite and sex.

Table S14. Multivariate analysis of association between metabolites in polyamine synthesis and OS and RFS by sex.

Variable[a]	OS				RFS			
	Females		Males		Females		Males	
	HR (95% CI)	*P* value	HR (95% CI)	*P* value	HR (95% CI)	*P* value	HR (95% CI)	*P* value
N^1, N^{12}-diacetylspermine (DAS)	2.45 (0.96–6.22)	0.059	0.66 (0.42–1.02)	0.060	1.10 (0.50–2.42)	0.813	1.07 (0.61–1.90)	0.812
Spermidine	0.51 (0.15–1.67)	0.264	3.21 (1.04–9.93)	0.043	0.28 (0.09–0.91)	0.034	4.18 (1.03–16.96)	0.045
Spermine	3.88 (1.50–10.06)	0.005	0.71 (0.43–1.18)	0.184	1.61 (0.90–2.88)	0.106	0.56 (0.32–1.00)	0.048
N^1-acetylspermine	0.37 (0.16–0.86)	0.020	1.14 (0.74–1.75)	0.557	0.92 (0.46–1.83)	0.816	1.62 (0.99–2.63)	0.054
Ornithine	0.64 (0.10–4.28)	0.646	0.78 (0.40–1.55)	0.481	0.41 (0.10–1.67)	0.213	0.86 (0.45–1.67)	0.660
Arginine	5.63 (1.33–23.78)	0.019	1.06 (0.54–2.08)	0.869	3.22 (1.14–9.13)	0.028	1.57 (0.74–3.34)	0.237
Citrulline	2.57 (1.04–6.33)	0.040	0.73 (0.47–1.15)	0.178	1.24 (0.60–2.59)	0.561	0.88 (0.51–1.50)	0.630
Argininosuccinic acid	0.57 (0.23–1.38)	0.212	0.74 (0.47–1.19)	0.214	1.20 (0.53–2.73)	0.667	0.43 (0.24–0.78)	0.005
Anatomic location = RCC (ref: LCC)	0.33 (0.06–1.69)	0.182	0.80 (0.31–2.08)	0.648	0.55 (0.14–2.08)	0.375	0.50 (0.15–1.69)	0.265
Clinical stage = late (ref: early)	119.96 (4.62–3116.09)	0.004	3.56 (0.94–13.45)	0.061	1.27 (0.19–8.70)	0.805	0.95 (0.20–4.60)	0.947
Chemotherapy = Yes (ref: No)	0.10 (0.01–1.50)	0.096	2.20 (0.56–8.66)	0.258	4.54 (0.59–34.72)	0.145	1.14 (0.21–6.14)	0.878
Age	1.18 (1.05–1.33)	0.007	1.15 (1.07–1.23)	<0.001	0.97 (0.87–1.08)	0.625	0.90 (0.82–0.98)	0.014

[a] All the variables listed were included in one multivariate Cox PH model. The abundance of each metabolite was treated as a continuous variable and was $\log_2$ transformed.
